# Supplementary material for: PI3K/AKT/β-Catenin Signaling Regulates Vestigial-Like 1 Which Predicts Poor Prognosis and Enhances Malignant Phenotype in Gastric Cancer
Source: Cancers (Basel). 2019 Dec 3;11(12):1923. doi: 10.3390/cancers11121923 (PMC6966677; doi:10.3390/cancers11121923)
Supplement: Supplementary file 1 [file cancers-11-01923-s001.pdf]

cancers

MDPI

Article

PI3K/AKT/β-Catenin Signaling Regulates Vestigial-Like 1 Which Predicts Poor Prognosis and Enhances Malignant Phenotype in Gastric Cancer

Bo-Kyung Kim [1,†,*], Jae-Ho Cheong [2,3,†], Joo-Young Im [1], Hyun Seung Ban [4], Seon-Kyu Kim [1], Mi-Jung Kang [1], Jungwoon Lee [5], Seon-Young Kim [1,6], Kyung-Chan Park [1,6], Soonmyung Paik [3], and Misun Won [1,6,*]

Table S1. Expression of VGLL1 and clinicopathologiccharacteristics of 556 patients with gastric cancer.

		VGLL1 low (n = 252)	VGLL1 high (n = 304)	Chi-square (p-value)
**Age**	<60	125 (49.4%)	128 (50.6%)	0.069
	>60	127 (41.9%)	176 (58.1%)	
Sex	Male	173 (45.5%)	207 (54.5%)	0.887
	Female	79 (44.9%)	97 (55.1%)	
Lauren	Intestinal	92 (38.5%)	147 (61.5%)	0.000368
	Diffuse	112 (56.9%)	85 (43.1%)	
	Mixed	14 (58.3%)	10 (41.7%)	
Tumor size	<100	224 (45.4%)	269 (54.6%)	0.881
	>100	28 (44.4%)	35 (55.6%)	
pT stage	T1	3 (20%)	12 (80%)	0.00001
	T2	13 (28.9%)	32 (71.1%)	
	T3	50 (72.5%)	19 (27.5%)	
	T4	186 (43.6%)	241 (56.4%)	
pN stage	N0	61 (57%)	46 (43%)	0.0418
	N1	45 (40.2%)	67 (59.8%)	
	N2	61 (45.9%)	72 (54.1%)	
	N3	85 (41.7%)	119 (58.3%)	
TMN stage	I	4 (19%)	17 (81%)	0.000017
	II	88 (60.7%)	57 (39.3%)	
	III	155 (41.9%)	215 (58.1%)	
	IV	5 (25%)	15 (75%)	
AJCC stage	I	13 (32.5%)	27 (57.5%)	0.000645
	II	95 (58.6%)	67 (41.4%)	
	III	122 (41.6%)	171 (58.4%)	
	IV	22 (36.1%)	39 (63.9%)	
Lympatic invasion	Absent	60 (63.2%)	35 (36.8%)	0.003371
	Present	119 (45.6%)	142 (54.4%)	
Vein invasion	Absent	71 (55.9%)	56 (44.1%)	0.1783
	Present	106 (48.6%)	113 (51.4%)	
Neural invasion	Absent	51 (58%)	37 (42%)	0.1815
	Present	83 (66.9%)	41 (33.1%)	

pT, primary tumor; pN, regional lymph nodes.

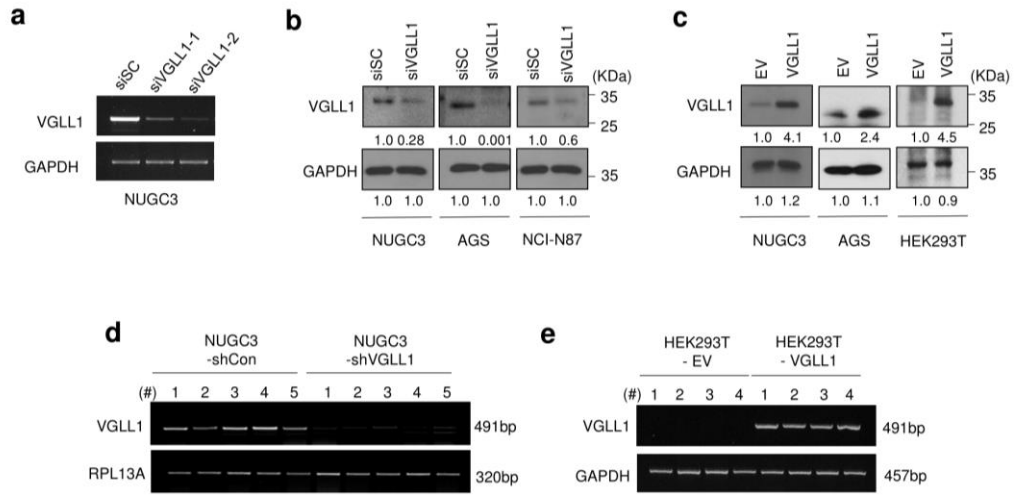

Figure S1. The expression of VGLL1. (**a**,**b**) The cells were transfected with siScramble or siVGLL1. (**c**) The cells were transfected with pcDNA3 or pcDNA3-VGLL1. The expression of VGLL1 was analyzed by western blotting. (**d**,**e**) VGLL1 expression in tumor tissues of the xenograft model as measured byRT-PCR.

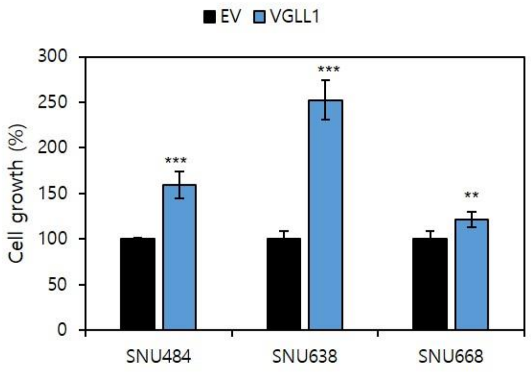

Figure S2. The effect of VGLL1 overexpression on the proliferation of gastric cancer cells expressing low VGLL1. SNU484, SNU638, and SNU668 cells were transfected with pcDNA3.1 or pcDNA3.1–VGLL1 and incubated for 72 h. Total cells were analyzed by Sulforhodamine B staining.

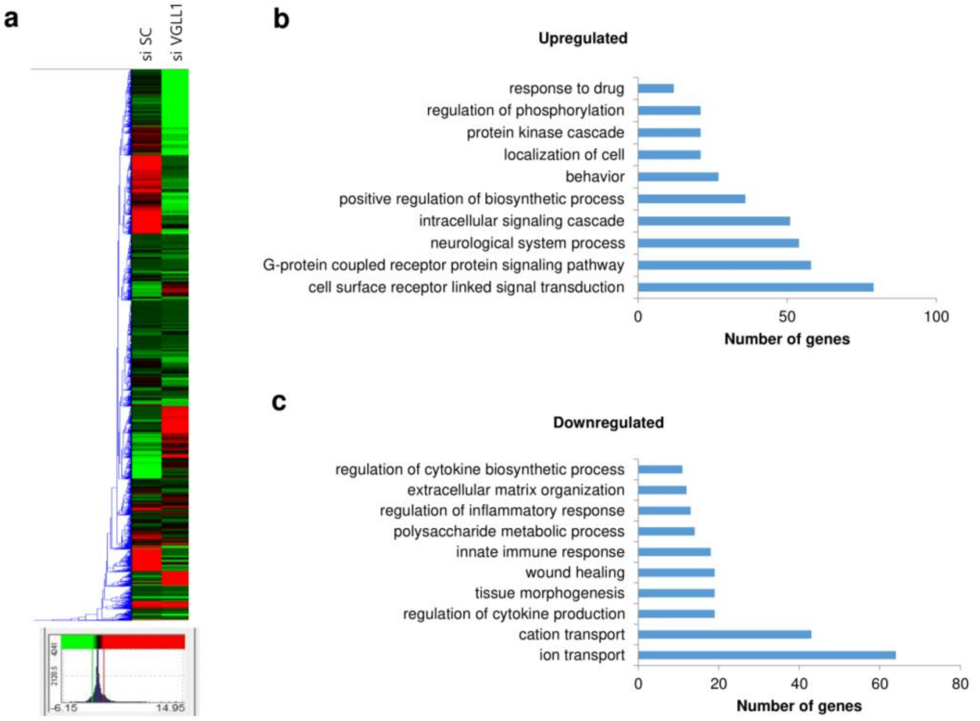

d

LogFC	GeneSymbol	Description
-6.75	CXCR7	*Homo sapiens* chemokine (C-X-C motif) receptor 7 (CXCR7)
-6.67	ITIH2	*Homo sapiens* inter-alpha-trypsin inhibitor heavy chain 2 (ITIH2)
-4.65	CXCL12	*Homo sapiens* chemokine (C-X-C motif) ligand 12 (CXCL12), transcript variant 2
-4.46	VGLL1	*Homo sapiens* vestigial like 1 (Drosophila) (VGLL1)
-4.11	FGA	*Homo sapiens* fibrinogen alpha chain (FGA), transcript variant alpha
-2.99	CSF3	*Homo sapiens* colony stimulating factor 3 (granulocyte) (CSF3), transcript variant 1
-2.83	IL6	interleukin 6 (interferon, beta 2)
-2.73	MMP9	*Homo sapiens* matrix metallopeptidase 9 (gelatinase B, 92kDa gelatinase, 92kDa type IV collagenase) (MMP9)
-2.35	NODAL	*Homo sapiens* nodal homolog (mouse) (NODAL)
-2.31	CD69	CD69 molecule [Source:HGNC Symbol;Acc:1694] [ENST00000416624]
-1.64	MMP1	*Homo sapiens* matrix metallopeptidase 1 (interstitial collagenase) (MMP1), transcript variant 1
-1.57	MMP7	*Homo sapiens* matrix metallopeptidase 7 (matrilysin, uterine) (MMP7)
-1.29	ITGA1	*Homo sapiens* integrin, alpha 1 (ITGA1)

Figure S3. Microarr ayanalysis of NUGC3 cells treated with siVGLL1. (**a**) Heatmapanalysis. (**b**,b) Gene Ontology pathway analysis. Gene set enrichment analyses were performed using the DAVID bioinformatics. (**d**) The list of down-regulated genes after VGLL1 knockdown.

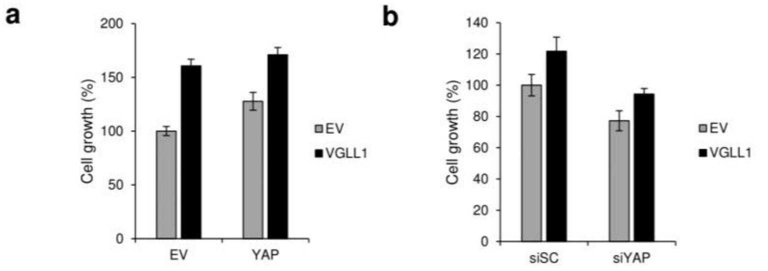

Figure S4. Effect of YAP knockdown/overexpression on cell growth in NUGC3-EV and NUGC3-VGLL1 cells. (**a**) NUGC3-EV and NUGC3-VGLL1 cells were transfected with pcDNA3.1 (EV) or pcDNA3.1-Myc-YAP. Cell growth was measured by live-cell imaging (IncuCyteZOOMsystem). (**b**) NUGC3-EV and NUGC3-VGLL1 cells were treated with 20 nM siScramble (SC) or siYAP. Cell growth was measured by live-cell imaging (IncuCyteZOOMsystem).
